# Asymptomatic Carotid Stenosis: Intervention or Best Medical Therapy?

**DOI:** 10.1007/s11910-018-0888-5

**Published:** 2018-09-24

**Authors:** Kamran Gaba, Peter A Ringleb, Alison Halliday

**Affiliations:** 1 Nuffield Department of Population Health, Medical Research Council Population Health Research Unit, University of Oxford, Richard Doll Building, Old Road Campus, Oxford, OX3 7LF UK; 2 Nuffield Department of Population Health, Clinical Trials Service Unit and Epidemiological Studies Unit, University of Oxford, Oxford, UK; 3 Department of Neurology, Heidelberg University Hospital, Heidelberg, Germany; 4 Nuffield Department of Surgical Sciences, University of Oxford, Oxford, UK

**Keywords:** Carotid endarterectomy, Carotid artery stenting, Best medical therapy, Asymptomatic, Carotid artery stenosis

## Abstract

**Purpose of Review:**

Provide a current overview regarding the optimal strategy for managing patients with asymptomatic carotid artery stenosis.

**Recent Findings:**

Carotid endarterectomy (CEA) and carotid artery stenting (CAS) reduce long-term stroke risk in asymptomatic patients. However, CAS is associated with a higher risk of peri-procedural stroke. Improvements in best medical therapy (BMT) have renewed uncertainty regarding the extent to which results from older randomised controlled trials (RCTs) comparing outcomes following carotid intervention can be generalised to modern medical practise.

**Summary:**

‘Average surgical risk’ patients with an asymptomatic carotid artery stenosis of 60–99% and increased risk of late stroke should be considered for either CEA or CAS. In patients deemed ‘high risk’ for surgery, CAS is indicated. Use of an anti-platelet, anti-hypertensive and statin, with strict glycaemic control, is recommended. Results from ongoing large, multicentre RCTs comparing CEA, CAS and BMT will provide clarity regarding the optimal management of patients with asymptomatic carotid artery stenosis.

## Introduction

Thromboembolic stroke is a major cause of morbidity and mortality in the United Kingdom (UK). It has been reported that, annually, more than 100,000 people have a stroke and 12.5% die within 30 days [1]. Strokes are the fourth largest cause of death and a substantial cause of disability in British adults [1]. In the recent Global Burden of Disease study, the number of global lost disability-adjusted life years (DALYs) due to cerebrovascular disease had increased by 18.9% from 1990 [2]. Indeed, by 2010, cerebrovascular disease was the third most common cause of lost DALY globally (compared to fifth most common in 1990) [2]. In the United States of America (USA) alone, strokes are the third most common cause of death, with atherosclerotic stenosis of the carotid artery implicated in 20–25% of all strokes [3]. Even when asymptomatic, stenosis of the carotid artery has been reported to place an individual at more than 3% increased risk of having a stroke in the next year (a greater than 50% increased relative risk) [4].

Carotid endarterectomy (CEA) and carotid artery stenting (CAS) are effective long-term stroke prevention strategies in symptomatic patients. However, uncertainty still remains regarding the optimal technique for long-term prevention of vascular events in asymptomatic patients and, indeed, whether either is sufficiently better than best medical therapy (BMT). This review article outlines the techniques of carotid intervention, presents the evidence for and against the use of CEA and CAS in stroke prevention in asymptomatic patients, summarises the role of BMT and highlights the current research focus in this important subject.

## CEA: the Technique

CEA can be performed under local anaesthesia (LA) or under general anaesthesia (GA) with or without the use of a shunt. The General Anaesthesia versus Local Anaesthesia for Carotid surgery (GALA) trial reported no difference in stroke or mortality rates between the two types of anaesthesia in patients undergoing CEA [5]. The operation begins with an oblique incision, following the anterior border of the sternocleidomastoid muscle [6]. This is then retracted and the carotid bifurcation is dissected out. Heparin is administered intravenously and the internal, external and common carotid arteries are clamped [6]. A longitudinal arteriotomy is made, extending from the common carotid artery into the internal carotid artery [6]. The plaque is removed and the artery repaired. The repair may include either primary closure of the vessel or placement of a patch to widen the vessel or an eversion CEA may be performed [6]. Similar outcomes (relating to peri-operative major stroke and death rate) have been described between eversion and conventional CEA methods in the Eversion Carotid Endarterectomy versus Standard Trial (EVEREST) randomised clinical trial (RCT) [7]. The incidence of early carotid artery occlusion and re-stenosis at mean follow-up of 14.9 months was also similar between the two groups [7].

However, performing CEA also has local risks: cranial nerve injury, peri-operative myocardial infarction (MI) and post-operative haematoma are well-recognised potential complications [8]. Moreover, patients with concurrent coronary artery disease and contralateral carotid artery occlusion are considered high risk for CEA [9, 10]. Other factors that make CEA more technically difficult include a high carotid bifurcation, recurrent stenosis after previous CEA, contralateral cranial nerve palsies, radical neck dissection and previous radiation to the area [8]. In these patients, CAS may be a feasible alternative [11–13].

## CAS: the Technique, Indications and Challenges

CAS is usually carried out under LA. A catheter is inserted percutaneously into the common femoral artery (or rarely via a brachial or even direct carotid access) and advanced until the common carotid artery is reached [6]. A guidewire is deployed across the stenosis so that a stent can be placed and a balloon may be used to expand the stent [6]. Embolic protection devices (EPDs) are often used to try to minimise embolisation of atherosclerotic material distal to the stenosis, preventing neurological ischaemia [6]. Carotid flow reversal can also be used to minimise the peri-procedural stroke risk.

CAS is less invasive than CEA and it may be performed in patients with co-morbidities that confer an unjustifiably high surgical risk for CEA whilst being able to potentially treat lesions that are inaccessible by surgery. However, various studies have shown that CAS may carry a higher stroke risk in symptomatic patients compared to asymptomatic patients [14, 15].

Registry data (as well as post hoc analysis of the RCTs comparing CAS and CEA in symptomatic patients) demonstrate that increased age is an independent predictor of poor outcome after CAS [15, 16]. This may be due to atherosclerotic disease being more advanced in old age, carotid vessels being more tortuous and plaques in the aortic arch being commoner. Engaging the common carotid artery may be technically challenging, causing distal embolisation of plaque and subsequent stroke or death.

Similarly to CEA, a learning curve also exists in acquiring the skills necessary to perform CAS. The Pro-CAS registry demonstrated a positive volume-outcome relationship: in centres performing less than 50 CAS procedures per year, the stroke or death rate was higher (at 5.9%) compared with centres performing more than 150 CAS procedures per year, whose stroke or death rate was 3.0% [14]. A similar relationship was demonstrated in the Stent-Protected Angioplasty versus Carotid Endarterectomy-1 (SPACE-1) study: centres performing less than 50 CAS procedures per year had a stroke or mortality rate of 4.6% whereas it was 2.9% in centres performing more than 50 CAS procedures per year [17]. This was also supported by the Carotid Stenting Trialists’ Collaboration (CSTC) who pooled data from three randomised trials: the Endarterectomy Versus Angioplasty in patients with Symptomatic Severe Carotid Stenosis (EVA-3S) trial, the aforementioned SPACE-1 trial and the International Carotid Stenting Study (ICSS) [18–21]. The CSTC group reported that the 30-day risk of stroke or death was not related to operator lifetime experience but it was higher in patients treated by interventionalists who performed less than 6 CAS procedures per annum [18].

## CEA in Asymptomatic Patients: the Evidence

Major RCTs comparing CEA versus BMT in asymptomatic patients are the Veterans Affairs (VA) Cooperative Study, the Asymptomatic Carotid Atherosclerosis Trial (ACAS) and the Asymptomatic Carotid Surgery Trial-1 (ACST-1), randomising patients between the 1980s and early 2000s [22–24]. The VA study (*n* = 444) reported an absolute risk reduction (ARR) in ipsilateral neurological events of 12.6% after CEA compared to BMT (8.0% versus 20.6% respectively; *p* < 0.01), in patients with a mean follow-up of almost 4 years [22]. However, there was no difference in the combined endpoint of stroke and death [22]. The ACAS study (*n* = 1662) reported that CEA reduced the 5-year risk of the combined endpoint of ipsilateral stroke and any peri-operative stroke or death by 53% compared to BMT (5.1% versus 11.0% respectively; *p* = 0.004) [23]. The ACST-1 (*n* = 3120) reported an ARR in stroke risk (when including peri-operative risks) of 4.1% at 5 years (CEA 6.9%, BMT 10.9%; *p* = 0.0001) and 4.6% at 10 years (CEA 13.4%, BMT 17.9%; *p* = 0.009) [24, 25]. This reduction in stroke risk was slightly improved when peri-operative events were excluded and half the reduction was in disabling and fatal strokes [25]. However, effective BMT was not available when these RCTs were undertaken.

The main findings of the above RCTs are summarised in Table 1.

**Table 1:** Summary of the evidence for the role of CEA in long-term stroke prevention in asymptomatic patients

Clinical trial	Years of recruitment	Degree of stenosis	Number of patients	Follow-up (years)	Findings
VA	1983–1987	≥ 50%	444	4.0	Ipsilateral TIA, amaurosis fugax or stroke
					CEA 8.0%, BMT 20.6%; *p* < 0.001
ACAS	1987–1993	≥ 60%	1662	2.7	Peri-procedural stroke or death, and post-operative ipsilateral stroke
					CEA 5.1%, BMT 11.0%; *p* = 0.004
ACST-1	1993–2003	≥ 60%	3120	5.0	Any stroke or peri-operative death
					CEA 6.9%, BMT 10.9%; *p* = 0.0001
				10.0	Any stroke or peri-operative death
					CEA 13.4%, BMT 17.9%; *p* = 0.009

*Abbreviations*: *ACAS* Asymptomatic Carotid Atherosclerosis Study, *ACST-1* Asymptomatic Carotid Surgery Trial 1, *BMT* best medical therapy, *CEA* carotid endarterectomy, *TIA* transient ischaemic attack, *VA* Veterans Affairs Cooperative Study

## CAS in Asymptomatic Patients: the Evidence

Unfortunately, the RCTs carried out comparing CEA with CAS have produced unreliable results due to heterogeneous patient populations, different endpoints being used, a variety of endovascular devices being used, varying use of EPDs between studies and varied endovascular experience of interventionalists participating in the RCTs.

The Stenting and Angioplasty with Protection in Patients at High Risk for Endarterectomy (SAPPHIRE) trial compared CAS with CEA in 334 patients at high operative risk [26]. This RCT included both symptomatic (*n* = 97) and asymptomatic (*n* = 237) patients [26]. Overall, there was no statistically significant difference in the composite endpoint of death, stroke or MI at 30 days with fewer CAS patients receiving re-intervention at 1 year than those undergoing CEA [26]. In asymptomatic patients specifically, the cumulative incidence of death, stroke or MI at 30 days was 5.4% for those receiving CAS and 10.2% undergoing CEA (*p* = 0.20) [26]. The equivalent outcome at 1 year was 9.9% in the CAS group and 21.5% in the CEA group and was statistically significant (*p* = 0.02) [26]. This is likely to be explained by the SAPPHIRE population having greater co-morbidities (75.5% of patients undergoing CEA had coronary artery disease), leading to a significantly higher rate of MI in the CEA compared to the CAS group (*p* = 0.03) [26]. At 3 years, stroke rates were comparable between asymptomatic patients undergoing CEA and CAS (9.2 and 10.3% respectively) whilst the combined endpoint of death, stroke or MI was also similar (29.2% versus 21.4% respectively) [27].

The Carotid Revascularisation Endarterectomy versus Stenting Trial-1 (CREST-1) was a large multicentre RCT, including 1321 symptomatic and 1181 asymptomatic patients [28]. Each surgeon and interventionalist was required to meet a minimum set of standards for experience and performance whilst EPDs were used in 96.1% of patients [28]. Overall, there was no statistically significant difference in the rates of the combined endpoint of stroke, MI or death within 30 days between CAS and CEA [28]. In asymptomatic patients, the combined endpoint at 30 days was 3.5% after CAS and 3.6% following CEA (hazard ratio (HR) 1.02 (0.55–1.86); *p* = 0.96) [28]. The peri-procedural stroke rate in asymptomatic patients undergoing CEA and CAS was 1.4% and 2.5% respectively (HR 1.88 (0.79–4.42); *p* = 0.15) [28]. Whilst the overall peri-procedural incidence of stroke or death was statistically significantly higher after CAS than after CEA in symptomatic patients (*p* = 0.02), this was not statistically significant for asymptomatic patients (*p* = 0.15) [28]. There were also more MIs following CEA in symptomatic than asymptomatic patients [28]. At 2 years, there was no statistically significant difference in the composite outcome of re-stenosis or re-occlusion and, at 4 years, there was no difference in ipsilateral stroke or death rate between CAS and CEA groups [28]. In asymptomatic patients specifically, the 4-year rate of stroke or death was 4.5% after CAS and 2.7% following CEA (*p* = 0.07) [28]. By 5 years, the rate of stroke in asymptomatic patients was 2.5% after CAS and 2.7% following CEA [29••]. Increased age, female sex, hypertension, diabetes and dyslipidaemia were independent predictors of re-stenosis or re-occlusion at 2 years after CAS whilst smoking was statistically significantly associated with re-stenosis after CEA [30]. At 10 years of follow-up, there was no statistically significant difference in the composite endpoint of stroke, MI or death and subsequent ipsilateral stroke between CAS and CEA groups in either symptomatic or asymptomatic subgroups [29••]. In asymptomatic patients, the rates of post-procedural ipsilateral strokes were similar at 5 and 10 years, regardless of the modality of revascularisation used [29••]. Therefore, symptom status may predict peri-procedural risk of complications but does not appear to be relevant in the long term.

The Asymptomatic Carotid Trial-1 (ACT-1) reported that CAS was non-inferior to CEA with regard to the composite endpoint of death, stroke or MI within 30 days of the procedure in 1453 asymptomatic patients with severe carotid artery stenosis (3.3% versus 2.6% respectively; *p* = 0.60) [31••]. Peri-procedural death was equivalent between CAS and CEA groups (0.1% versus 0.3% respectively; *p* = 0.60), as was peri-procedural MI (0.5% versus 0.9% respectively; *p* = 0.41) [31••]. CEA halved the risk of peri-procedural stroke compared to CAS, though this was not statistically significant (1.4% versus 2.8% respectively; *p* = 0.23) [31••]. By 1-year follow-up, 3.8% of patients receiving CAS and 3.4% of patients undergoing CEA had died or experienced a stroke or MI and, by 5 years, the ipsilateral stroke rate was 2.2% after CAS and 2.7% following CEA (*p* = 0.51) [31••]. This RCT reported its findings recently and broadly supports the findings of CREST-1. However, its results may not be fully generalisable to contemporary practise as they only used one type of stent and EPD [28].

The main findings of the above RCTs are summarised in Table 2.

**Table 2:** Summary of the evidence for the role of CAS in long-term stroke prevention, compared to CEA, in asymptomatic patients

Clinical trial	Years of recruitment	Number of patients	Follow-up (years)	Findings
SAPPHIRE*	2000–2002	237	3.0	Peri-procedural MI, stroke, death and post-procedural ipsilateral stroke and death
				CEA 29.2%, CAS 21.4%
CREST-1*	2000–2008	1181	5.0	Peri-procedural MI, stroke, death and post-procedural ipsilateral stroke
				CEA 5.4%, CAS 6.1%; *p* = 0.95
			10.0	Peri-procedural MI, stroke, death and post-procedural ipsilateral stroke
				CEA 10.1%, CAS 9.6%
ACT-1	2005–2013	1453	5.0	Post-procedural ipsilateral stroke
				CEA 2.7%, CAS 2.2%; *p* = 0.51

*Abbreviations*: *ACT-1* Asymptomatic Carotid Stenosis 1, *CAS* carotid artery stenting, *CEA* carotid endarterectomy, *CREST-1* Carotid Revascularisation Endarterectomy versus Stenting Trial, *MI* myocardial infarction, *SAPPHIRE* Stenting and Angioplasty with Protection in Patients at High Risk for Endarterectomy

*Subgroup analysis

## BMT in Asymptomatic Patients: Recommendations

Many RCTs listed above were carried out before the advancement of modern medical therapies (e.g. widespread prescribing of statins) and, as such, it is unclear what effect this might have on outcomes. Most patients with carotid artery stenosis today should be prescribed a statin, anti-platelet agent and a blood pressure-modifying agent [32••]. However, in the early years of recruitment in the ACST-1, less than 10% of patients were taking lipid-lowering therapy at baseline [25]. This increased to 80% by the end of follow-up [25]. This could have potentially confounded outcomes as it has been demonstrated that a 1-mmol L^−1^ reduction in low-density lipoprotein (LDL) cholesterol reduces the 5-year risk of stroke by approximately 25% [33–35]. This might explain why statin use was associated with a halving of the risk of stroke following CEA in the ACST-1 [24, 25]. However, irrespective of statin use, CEA still halved the stroke rate in the ACST-1 [24, 25].

The recent guidelines published by the European Society for Vascular Surgery (ESVS) in 2018 recommend that a healthy diet, smoking cessation and physical activity should all be instituted for risk factor reduction in patients with known asymptomatic carotid artery stenosis [36••]. Low-dose aspirin was also recommended as the anti-platelet agent of choice despite a lack of adequately powered studies (with insufficient follow-up) showing benefits of anti-platelet agents in patients with asymptomatic carotid artery stenosis and conflicting results reported in RCTs [37, 38, 39••, 40]. Patients who are intolerant of aspirin should be prescribed clopidogrel instead [36••]. Anti-platelet therapy should be used peri-procedurally and long-term following both CEA and CAS, with dual anti-platelet therapy recommended for at least 1 month post-CAS [36••].

Lipid-lowering therapy in the form of statins has also been recommended for long-term prevention of cardiovascular disease in asymptomatic patients [36••]. This is evidence-based as a Cochrane review demonstrated a significant reduction in all-cause mortality, fatal/non-fatal strokes and revascularisation in patients randomised to statins [41]. The pharmacological agents that have been widely prescribed in RCTs are atorvastatin and rosuvastatin and attempts should be made to achieve targets of either a 50% reduction in LDL cholesterol or a level less than 1.8 mmol L^−1^ (70 mg dL^−1^) [42–44].

Whilst the effect of anti-hypertensive therapy on stroke prevention in patients with asymptomatic carotid artery stenosis has not been formally evaluated, treatment of high blood pressure is associated with reduction and regression of carotid artery stenoses [45]. Indeed, a meta-analysis demonstrated a reduction in stroke risk that was proportional to lowering of systolic blood pressure and a Chinese RCT reported reduced stroke risk associated with use of enalapril [46, 47•]. In reality, the type of pharmacological agent used is less important than achieving the target of reducing blood pressure below 140/90 mm Hg in non-diabetic patients with asymptomatic carotid artery stenosis [48]. Blood pressure should be maintained below 180/90 mm Hg peri-procedurally following CEA and CAS [36••].

In diabetic patients with asymptomatic carotid artery stenosis, tight glycaemic control is recommended as these patients are at increased risk of stroke [49]. Therefore, it is important that risk factor reduction and BMT is appropriately prescribed in diabetic patients. In a study of type II diabetes patients, the use of lipid-lowering therapy, anti-platelet agents and anti-hypertensive medications was associated with a 60% reduction in cardiovascular events and deaths [50]. Moreover, the UK Prospective Diabetes Study reported that intensive blood pressure control in diabetic patients reduced the relative risk of stroke by 44% compared to patients with a higher blood pressure [51]. The joint European Society for Hypertension/European Society of Cardiology guidelines (2013) recommend that the target blood pressure in diabetic patients with asymptomatic carotid artery stenosis should be less than 140/85 mm Hg [52].

## Other Considerations

Carotid intervention is now much safer than at any time in the past with CEA outcomes significantly improved when patients are operated on by vascular surgeons compared with cardiothoracic surgeons, general surgeons or neurosurgeons [53–55, 56•, 57–59]. This might simply reflect that vascular surgeons perform CEAs more frequently than other specialists and are more familiar with the operation. This positive volume-outcome relationship in CEA has been proven to reduce both mortality and combined mortality/stroke [60•]. This trend of sub-specialisation has not been reflected when vascular surgeons, interventional radiologists, neurosurgeons and interventional neuroradiologists performed CAS in the CREST-1 [28]. This was shown to affect outcomes, with vascular surgeons and interventional radiologists having worse CAS outcomes than interventional neuroradiologists or interventional cardiologists [61]. This may reflect the varying complexity of cases referred to each specialty or the lack of experience with catheter-based procedures in some specialties at the time of the CREST-1. By contrast, the ACAS employed such strict credentialing that 40% of all applicants were rejected from participating in the RCT due to an unfavourable safety record [23]. So, these RCT findings may not be generalisable to contemporaneous practise.

Use of EPDs may reduce the peri-procedural stroke rate following CAS. A systematic review reported a reduced 30-day death or stroke rate from 5.5 to 1.8% in patients undergoing CAS without and with EPDs respectively [62]. Data from a large registry have also confirmed the finding that EPDs reduce the death or stroke rate in patients undergoing CAS, with the use of EPDs being an independent protective factor [63]. Varying use of EPDs in the RCTs, in contrast to modern practise, must be taken into account when interpreting findings.

Novel medical therapies may also show benefit in reducing the long-term stroke risk in asymptomatic patients. A subgroup analysis of the recently completed Cardiovascular Outcomes for People using Anticoagulation Strategies (COMPASS) RCT showed that addition of low-dose rivaroxaban to aspirin (in 1919 patients with previous carotid artery revascularisation or asymptomatic carotid artery stenosis of at least 50%) reduced the overall major adverse cardiovascular event rate (HR 0.63 (0.38–1.05; *p* = 0.07) [64••]. This was not at the expense of an increased bleeding risk [64••]. Therefore, whilst this subgroup analysis was inadequately powered to provide meaningful conclusions, larger RCTs may reveal a significant effect of adding low-dose rivaroxaban to aspirin in long-term stroke prevention.

## Conclusions and Future Perspectives

It is clear that both CEA and CAS reduce the long-term stroke risk in asymptomatic patients. With regard to peri-procedural outcomes, a recent meta-analysis of 3901 asymptomatic patients randomly assigned to CEA (*n* = 1585) or CAS (*n* = 2316) reported that CAS was associated with a significantly higher risk of peri-procedural stroke than CEA (2.6% versus 1.3% respectively; *p* = 0.04) [65••]. This was largely driven by more minor strokes following CAS than CEA (2.2% versus 1.0%; *p* = 0.05) [65••]. Rates of death, major stroke, ipsilateral stroke and MI were comparable between CEA and CAS [65••].

ESVS guidelines (2018) therefore recommend that patients with an ‘average surgical risk’ and an asymptomatic carotid artery stenosis of 60–99% should be considered for CEA only in the presence of one or more characteristics that may be associated with an increased risk of late ipsilateral stroke [36••]. According to this guideline, imaging/clinical criteria that might confer an increased risk of stroke on BMT include silent infarction on computerised tomography, stenosis progression, large plaque area, plaque echolucency, intra-plaque haemorrhage on magnetic resonance imaging, impaired cerebral vasoreactivity, spontaneous embolisation on transcranial Doppler ultrasound and/or history of contralateral transient ischaemic attack [36••]. CAS is also a feasible alternative in these patients [36••]. However, in selected patients who are deemed ‘high risk’ for surgery with an asymptomatic carotid artery stenosis of 60–99%, CAS is indicated [36••]. Again, these patients must be deemed at increased risk of late ipsilateral stroke [36••]. Requirement for any intervention is a documented peri-procedural risk of stroke/death of less than 3% and the patient must have a life expectancy exceeding 5 years [36••]. These recommendations have also been supported by the European Society of Cardiology, who have reduced their threshold of acceptable complication rates following CEA and CAS from 6 to 3%, in line with the ESVS guidelines [44, 66••].

However, these guidelines are based on RCT findings which, due to the antiquated nature of the BMT and interventional techniques used, may not be generalisable to contemporary practise. There is therefore uncertainty regarding which technique is superior or, indeed, if advancements in BMT have replaced the need for performing carotid interventions.

In order to address this important issue, the Stent-protected Angioplasty in Asymptomatic Carotid Artery Stenosis versus Carotid Endarterectomy-2 (SPACE-2) trial was designed to compare BMT alone versus BMT with CEA versus BMT with CAS in asymptomatic patients. Unfortunately, however, due to slow recruitment of patients, the RCT was modified to compare BMT alone versus BMT with CEA and BMT alone versus BMT with CAS, with the initial results recently being reported [67••]. In 513 asymptomatic patients, there were no peri-procedural deaths recorded following CEA or CAS [67••]. This is the first RCT to report no peri-procedural deaths following CEA and CAS [67••]. There were also no deaths or strokes within 30 days of patients being randomised to BMT whilst the combined peri-procedural stroke and death rate was 2.0% after CEA and 2.5% following CAS [67••]. These results, whilst promising, have wide confidence intervals due to the small sample size and suggest a ‘missed opportunity’ to clarify uncertainty in this important subject. Long-term outcomes are awaited.

Ongoing RCTs such as the Carotid Revascularisation and Medical Management for Asymptomatic Carotid Stenosis trial (CREST-2) and the European Carotid Surgery Trial (ECST-2) will compare both CAS (with use of EPDs) and CEA with medical management in asymptomatic patients with severe carotid artery stenosis whilst the Endarterectomy Combined with Optimal Medical Therapy (OMT) versus OMT Alone in Patients With Asymptomatic Severe Atherosclerotic Carotid Artery Stenosis at Higher-than-average Risk of Ipsilateral Stroke (ACTRIS) trial will compare CEA, in conjunction with BMT, versus BMT alone [68••, 69••].

The Asymptomatic Carotid Surgery Trial-2 (ACST-2) will compare CAS with CEA for long-term stroke prevention in asymptomatic patients with severe carotid artery stenosis on BMT [70]. The ACST-2, due to complete recruitment by the end of 2019, is the largest RCT ever conducted comparing CAS with CEA and should provide reliable evidence as it is being conducted internationally with experienced collaborators and high-quality BMT in addition to modern carotid revascularisation strategies [70].

The results of these RCTs are eagerly anticipated and awaited in the 2020s as they will provide clarity, in a large number of patients (whilst also providing an opportunity for meta-analysis), regarding the effect of the increased use of statins, new stent designs and safer CAS techniques compared with CEA and BMT to better inform the management of patients with asymptomatic carotid artery stenosis.

## Abbreviations

ACAS: Asymptomatic Carotid Atherosclerosis Trial
ACST: Asymptomatic Carotid Surgery Trial
ACT: Asymptomatic Carotid Trial
ACTRIS: Endarterectomy Combined with Optimal Medical Therapy (OMT) versus OMT Alone in Patients With Asymptomatic Severe Atherosclerotic Carotid Artery Stenosis at Higher-than-average Risk of Ipsilateral Stroke
ARR: absolute risk reduction
BMT: best medical therapy
CAS: carotid artery stenting
CEA: carotid endarterectomy
COMPASS: Cardiovascular Outcomes for People using Anticoagulation Strategies
CREST: Carotid Revascularisation Endarterectomy versus Stenting Trial
CSTC: Carotid Stenting Trialists’ Collaboration
DALY: disability-adjusted life year
ECST: European Carotid Surgery Trial
EPD: embolic protection device
ESVS: European Society for Vascular Surgery
EVA-3S: Endarterectomy Versus Angioplasty in patients with Symptomatic Severe Carotid Stenosis
EVEREST: Eversion Carotid Endarterectomy versus Standard Trial
GA: general anaesthesia
GALA: General Anaesthesia versus Local Anaesthesia for Carotid surgery
ICSS: International Carotid Stenting Study
LA: local anaesthesia
LDL: low-density lipoprotein
MI: myocardial infarction
OMT: optimal medical therapy
RCT: randomised clinical trial
SAPPHIRE: Stenting and Angioplasty with Protection in Patients at High Risk for Endarterectomy
SPACE: Stent-Protected Angioplasty versus Carotid Endarterectomy
UK: United Kingdom
USA: United States of America
VA: Veterans Affairs
